# Microbial community assembly in the fermentation of cigar tobacco leaves

**DOI:** 10.3389/fbioe.2026.1779182

**Published:** 2026-04-01

**Authors:** Tianfei Zheng, Dongfeng Guo, Yaqi Shi, Jinlong Zhou, Kun Zong, Naihong Ding, Xingjiang Li

**Affiliations:** 1 Technology center, China Tobacco Anhui Industrial Co. Ltd., Hefei, Anhui, China; 2 School of Food and Biological Engineering, Hefei University of Technology, Hefei, Anhui, China

**Keywords:** cigar tobacco leaves, community assembly, fermentation, microbial community, quorum sensing

## Abstract

Microbial communities play pivotal roles in the fermentation of cigar tobacco leaves. Although high-throughput sequencing technology has facilitated the exploration of these communities, a comprehensive understanding of their assembly mechanisms remains elusive. This review integrates the current knowledge regarding microbial sources, ecological dynamics, and evolutionary processes during cigar tobacco fermentation. We systematically assess the abiotic factors (temperature, humidity, nutrients) and biotic interactions (quorum sensing, metabolic coordination) which influence the microbial community. Moreover, we put forward strategies for synthetic community engineering and discuss the emerging applications of artificial intelligence in fermentation optimization. These insights deepen the understanding of microbial communities in cigar tobacco leaves, and provide new perspectives on regulating microbial communities to enhance the fermentation quality of cigar tobacco leaves.

## 1 Introduction

Cigar is a tobacco product rolled from dry and fermented tobacco leaves, and the fermentation of cigar tobacco leaves is a crucial process that enhances the appearance, color, physical properties, aroma concentration and quality of cigar leaves. Compared with the high-temperature processing of flue-cured tobacco, the fermentation process of cigar tobacco leaves is milder, more time-consuming and dominated by natural microbial metabolism (Zhang et al, 2024a; Zhang et al., 2023b). In this process, the microbial community in cigar tobacco leaves plays a vital and complex role, and the formation of unique cigar aroma is a comprehensive result of multiple factors, including the intrinsic chemical components of cigar tobacco leaves (e.g., cellulose, proteins, terpenoids, nicotine), air drying and microbial fermentation. Among them, microbial fermentation is the key regulatory link connecting raw material characteristics and final flavor quality.

Microorganisms perform a dual role in the fermentation process of cigar tobacco leaves, including degrading macromolecules into small-molecule aroma precursors and facilitating their conversion into flavor compounds. Cellulases and hemicellulases secreted by microorganisms break down cell wall components (Zhao et al., 2025), improving processability, combustibility, and hygroscopy. Proteases hydrolyze proteins into amino acids, which either participate in non-enzymatic browning to form melanoidins, aldehydes, and ketones, or are metabolized into ammonia and amines that modulate irritation and aroma. Lipases degrade lipids into fatty acids, which oxidize into volatile aldehydes, ketones, and alcohols. Furthermore, microbial enzymes degrade chlorophyll and carotenoids (Moreira et al., 2024), with chlorophyll breakdown altering leaf color from green to yellow-brown. Polyphenol oxidase catalyzes the oxidation of phenolics (e.g., catechin to o-quinone), whose subsequent polymerization forms dark, aromatic pigments. These compounds not only uniform leaf color but also significantly enhance the complexity and fullness of cigar aroma.

Additionally, specific microorganisms synthesize characteristic aroma substances directly: yeast produces alcohols, esters and aldehydes via the glycolysis (EMP) pathway (Jiranek, 2024), with pyruvate converted to ethanol under anaerobic conditions and entering the TCA cycle aerobically; higher alcohols derive from amino acid degradation (e.g., leucine to isoamyl alcohol), and esters form via alcohol acyltransferase-catalyzed reactions between alcohols and acyl-CoA. Actinomycetes synthesize terpenoids through the MVA and MEP pathways (Maltseva et al., 2024), with terpene synthetases driving precursor cyclization and rearrangement to generate diverse terpenes. Carotenoid degradation yields β-ionone, damascenone and macrostigmatrienone (Mai et al., 2025), while ceptriatriene diol forms roasted-sweet solanone; monoterpenoids (geraniol, linalool, limonene) boost tobacco aroma freshness and head note, and sesquiterpenes (caryophyllene, farnesene) enhance aroma fullness, intensity and persistence (Usami, 2025).

The microbial community of cigar tobacco leaves stems from the uncontrolled external migration of microorganisms in the natural environment, and the composition and structure of microbial communities vary significantly in different regions due to differences in planting environment, tobacco variety and fermentation technology, which is the core reason for the diverse quality and aroma of cigar tobacco leaves from different producing areas (Zheng et al., 2022a). In Cuba, cigar tobacco leaves are often fermented via the natural stacking fermentation method (Stubbs, 2010). During the fermentation process, experienced workers regularly monitor the temperature and humidity changes inside the pile and conduct timely flipping operations to ensure uniform fermentation. Generally, the first fermentation may last for several months, followed by secondary fermentation and long-term natural aging fermentation to further improve quality. In Dominica, the fermentation of cigar tobacco leaves sometimes involves mixed fermentation combined with natural stacking fermentation and special techniques (Wang et al., 2024). Some manufacturers add natural microbial agents or fermentation promoters to accelerate the process and regulate the flavor direction, and use modern equipment for precise temperature and humidity control. Notably, partial Dominican cigar manufacturers adopt barrel fermentation with oak barrels containing residual wine/rum flavors (instead of new barrels) (Blanchard et al., 2001). Placing tobacco leaves in the enclosed wooden barrel environment, the barrel material (lignin, tannins) and residual flavor substances interact with tobacco leaves, endowing them with unique aromatic compounds and complex flavor layers, which is similar to wine-making flavor formation (Zhang et al, 2025a). The fermentation of cigar tobacco leaves in Honduras often adopts a segmented fermentation method: a rapid initial fermentation under high temperature and humidity induces basic biochemical changes, eliminating green impurities and harmful substances; then a slow subsequent fermentation under mild conditions further improves the quality. The fermentation conditions are adjusted in real time according to the stage of the cigar tobacco leaves to give full play to the characteristics of local tobacco leaves. Nicaraguan cigar tobacco fermentation combines traditional and modern methods. Based on traditional heap fermentation, advanced sensor devices and computer systems are used to monitor real-time parameters (temperature, humidity, oxygen content) and regulate the fermentation environment. Some manufacturers also screen and add local adaptive special strains to promote fermentation and flavor formation, retaining the stability of modern craftsmanship and the characteristics of traditional flavor.

Compared with world-famous cigar producing areas such as Cuba, the Dominica, and Nicaragua, the quality of cigar tobacco leaves produced in China is generally low due to the immature planting and fermentation technologies. To produce higher-quality cigar tobacco leaves through fermentation, researchers have conducted extensive work on studying the fermentation technology employed in the renowned cigar producing areas worldwide. With the exploration of the fermentation mechanism of cigar tobacco leaves, an increasing number of research has focused on the microbial community in cigar tobacco leaves. Initially, the culture-dependent method was used to isolate, culture and identify microorganisms in cigar tobacco leaves (Gong et al., 2009; Zhong et al., 2010). However, this method was time-consuming. With the widespread application of high-throughput sequencing technology, significant progress has been made in exploring the microbial communities in cigar tobacco leaves from different regions, varieties, or fermentation stages (Table 1). For example, Jia et al. found that *Staphylococcus* and *Aspergillus* are dominant in the bacterial and fungal communities during the fermentation of cigar tobacco leaves (Jia et al., 2023). Liu et al. found that in the bacterial community of cigar tobacco leaves, the relative abundances of *Ralstonia*, *Pseudomonas*, *Romboutsia*, *Chryseobacterium*, and *Comamonas* increased significantly after fermentation; in the fungal community, the relative abundances of *Aspergillus* and Phaeosphaeriaceae increased significantly, while that of *Cladosporium* decreased significantly (Liu et al, 2021a). Our previous studies have shown that among cigar tobacco leaves, the predominant genera are *Staphylococcus*, *Pseudomonas*, *Aspergillus*, *Sampaiozyma*, and *Alternaria*. However, their relative abundances vary significantly in different regions (Zheng et al., 2022a). For example, in terms of *Staphylococcus*, the abundance is 79.66% in Brazil and 12.56% in Indonesia. For *Pseudomonas*, it is 20.27% in Indonesia and 0.38% in Brazil. Regarding *Aspergillus*, it is 78.31% in Indonesia and 43.66% in China. And for *Sampaiozyma*, it is 17.67% in Brazil and 2.40% in Indonesia. Correspondingly, the characteristic aromatic components of cigar tobacco leaves in different producing areas also show obvious differences, which is the result of the combined action of tobacco variety, planting environment, microbial community, and fermentation technology, and microbial fermentation is the key factor for the formation of region-specific aromatic components. Indonesian cigar tobacco leaves are rich in (E)-6,10-dimethyl-5,9-undecadien-2-one, 4-(2,6,6-trimethyl-1-cyclohexen-1-yl)-3-buten-2-one, heptanal, and 1H-indole. The contents of pentanal and 2,4-dimethyl-benzaldehyde are high in Dominican cigar tobacco leaves. Chinese cigar tobacco leaves are rich in benzeneethanol, megastigmatrienone b, benzaldehyde, and b-damascone. Brazilian cigar tobacco leaves contain high contents of 6-methyl-5-hepten-2-one, 6,10-dimethyl-2-undecanone, 3-methyl-2-butenal, and 2,5-dimethyl-1H-pyrrole. These region-specific aromatic components are mostly the specific metabolic products of the dominant microbial genera in the corresponding producing areas, such as Indonesian (E)-6,10-dimethyl-5,9-undecadien-2-one is mainly synthesized by *Aspergillus*, and Brazilian 6-methyl-5-hepten-2-one is the metabolite of *Sampaiozyma*.

**TABLE 1:** Dominant microorganisms in cigar tobacco leaves from different producing areas or different fermentation stages.

Producing area	Processing stage	Dominant microorganisms
Brazil	Industrial fermentation	*Staphylococcus*, *Sampaiozyma* (Zheng et al., 2022a)
Indonesia	Industrial fermentation	*Pseudomonas*, *Aspergillus* (Zheng et al., 2022a)
Dominica	Industrial fermentation	*Staphylococcus*, *Aspergillus* (Zheng et al., 2022a)
Sichuan	Air drying	*Enterobacteriaceae* and *Alternaria* (Zhang et al., 2023b)
Sichuan	Agricultural fermentation	*Aquabacterium*, unclassified *Burkholderiaceae*, *Caulobacter*, *Brevundimonas*, and *Aspergillus* (Zhang et al., 2023b)
Sichuan	Industrial fermentation	*Acinetobacter*, *Staphylococcus*, *Aspergillus* (Si et al., 2023)
Yunan	Industrial fermentation	*Aspergillus*, *Alternaria*, and *Cladosporium* (Zhang et al., 2024a)

These fundamental studies facilitate the familiarization of the researchers regarding which bacteria, fungi, etc., exist in cigar tobacco leaves, as well as their functions and dynamic succession during the fermentation process. In recent years, a large number of studies have focused on the application of exogenous functional microbial strains and synthetic microbial communities (SynComs) in the fermentation of cigar tobacco leaves, and achieved positive results in improving fermentation quality. For example, Zhang et al. added a novel microbial fermentation medium produced by an edible medicinal fungus, *Tremella aurantialba* SCT-F3 (CGMCC No.23831) to improve the quality of cigar filler leaves (Zhang et al., 2023a). Guo et al. isolated and applied *Cyberlindnera fabianii* strains to effectively promote the degradation of complex organic substances and the formation of flavor precursors (Guo et al., 2024a); Yin et al. used starch-degrading bacteria to accelerate starch hydrolysis in tobacco leaves and regulate the microbial community structure (Yin et al., 2025); Pei et al. found that *Staphylococcus nepalensis* inoculation could significantly improve the volatile substance composition and sensory quality of cigar tobacco leaves (Pei et al., 2025). In addition, studies on pectin-degrading bacteria (Su et al., 2024), *Bacillus altitudinis* (Song et al., 2024), and engineered *Rhodotorula* strains (Guo et al., 2024b) have also confirmed the feasibility of exogenous microbial regulation in cigar fermentation.

However, the current research still has obvious gaps and limitations: (1) Most studies only focus on the effect of a single or several exogenous strains on fermentation quality, lacking systematic analysis of the intrinsic driving mechanisms of microbial community assembly after strain inoculation; (2) Few studies integrate microbial sources, ecological dynamics, and evolutionary processes to reveal the assembly rules and stability of cigar tobacco microbial communities; (3) The application of SynComs in cigar fermentation is still in the preliminary lab-scale stage, and the synergy/antagonism between the constructed strains and indigenous microbial communities, as well as the adaptability of SynComs in industrial fermentation environment, are still unclear; (4) The research on key functional microbial groups and their interaction with other microorganisms is not in-depth enough. At present, only a few studies have preliminarily explored the intrinsic driving mechanisms of microbial community formation after introducing exogenous microorganisms, and the comprehensive and systematic research on the assembly mechanism of cigar tobacco microbial community is still insufficient.

To promote the understanding of the microbial community in cigar tobacco leaves and provide theoretical support for improving the quality of Chinese cigar tobacco leaves, this review will investigate how microbial source, ecology, and evolution influence the assembly of the microbial community in cigar tobacco leaves. First, we introduce the fermentation characteristics of cigar tobacco leaves and the microbial sources. Next, we elaborate on the abiotic factors and biotic factors affecting microbial communities. Finally, we put forward strategies for reconstructing synthetic communities to enhance the quality of cigar tobacco leaves and discuss the potential applications of intelligent technologies, such as artificial sensors, spectroscopy, computer vision, machine learning, and big data analytics, in the fermentation processes of cigar tobacco leaves.

## 2 The fermentation characteristics of cigar tobacco leaves

The fermentation of cigar tobacco leaves resembles that of other solid-state fermented foods and beverages, such as cheeses, vegetables, Chinese Baijiu, and vinegar. These fermentations occur in an open environment (Song et al., 2020; Chai et al., 2021; Lu et al., 2018; Zhang et al., 2020). Under natural or manual control, various microorganisms and enzymes break down macromolecular substances into small molecular substances, and produce various aroma substances to improve the quality and flavor of cigar tobacco leaves (Banožić et al., 2020). After being harvested from the field, cigar tobacco leaves undergo drying, moisture regain, stacking fermentation, and flipping, which consist of three stages: air drying, agricultural fermentation, and industrial fermentation, among which air drying is the key pre-treatment stage for subsequent fermentation, and agricultural and industrial fermentation are the formal fermentation stages.

Air drying is a process in which fresh cigar tobacco leaves gradually dry in an appropriate temperature and humidity environment (Martínez-Martínez et al., 2012). During this process, the moisture content of fresh tobacco leaves drops from over 80% to below 20%, the color changes from green to yellow, and macromolecular substances (such as cellulose, pectin, and starch) degrade rapidly (Xing et al., 2025b). At this stage, fresh cigar tobacco leaves have just been detached from the tobacco plant and are still in a state of life activity, and their respiration consumes the stored substances rapidly (Wen et al., 2023; Yuan et al., 2023); meanwhile, microorganisms from the soil and air start to multiply and spread on the cigar tobacco leaves, and their metabolic activity accelerates the degradation of macromolecular substances. The synergy between plant endogenous enzymes (e.g., cellulase, protease) and initial microbial metabolism in the air-drying stage lays a foundation for the subsequent fermentation and aroma formation. The dominant microorganisms in the air-drying stage are mainly primary fermenters, such as *Pseudomonas*, *Sampaiozyma*, and *Alternaria*, which possess various carbohydrate-degrading enzymes that can utilize cellulose, hemicellulose, pectin, starch, or glycosides (Li et al, 2024a).

The agricultural fermentation process involves rewetting dried cigar tobacco leaves and then stacking them for fermentation (Zhang et al., 2023b). In this stage, the dried cigar tobacco leaves are rehydrated to a 25%–35% moisture content and then stacked together for fermentation. As the microorganisms grow, the temperature of the cigar tobacco leaves steadily increases by 2 °C–3 °C every day. Under the metabolic activity of microorganisms, cigar tobacco leaves undergo a complex biochemical process, and the monomers (such as glucose, amino acids, and fatty acids) produced by macromolecular degradation are transformed into various aromatic substances (such as alcohols, aldehydes, ketones, acids, esters). The microbial community in the agricultural fermentation stage is dominated by the primary fermenters from the air-drying stage, and the microbial diversity is relatively high, which is the key stage for the formation of basic aroma substances of cigar tobacco leaves.

Industrial fermentation is mainly used to make up for the deficiencies of agricultural fermentation, promoting the aroma and quality of tobacco to undergo an enhanced fermentation process (Zhang et al, 2024a). The metabolic activity of microorganisms promotes the accumulation of aroma substances and converts them into more complex aroma substances, increasing the variety and complexity of tobacco aroma. The dominant microorganisms in the industrial fermentation stage, such as *Acinetobacter*, *Staphylococcus*, and *Aspergillus*, play a significant role in the synthesis and transformation of aroma substances, making the aroma of tobacco more diverse and highlighting its fragrant characteristics. The industrial fermentation stage has higher requirements for environmental control, and the adjustment of temperature, humidity and oxygen content directly affects the succession of microbial community and the final flavor quality of cigar tobacco leaves.

## 3 Microbial sources in cigar tobacco leaves

**FIGURE 1:**
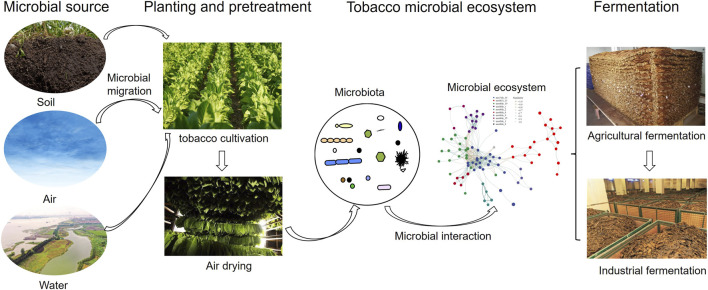
Schematic diagram of microbial ecosystem formation in cigar tobacco leaves and its role in fermentation. Microorganisms in cigar tobacco leaves migrate from soil, air, and water, colonize tobacco plants during the planting process, and further form a diverse microbial community with complex interactions during the air-drying process. This ecosystem then drives subsequent fermentation processes, including agricultural heap fermentation and industrial fermentation.

The microbial community in cigar tobacco leaves is influenced by both random and deterministic ecological processes (Liu et al., 2023). Random processes mainly consist of the random diffusion, birth, and death events of species. Deterministic processes, on the other hand, involve environmental filtering and interspecific interactions like competition, promotion, mutualism, and predation (Zhong et al., 2022). Different from other mass inoculated solid-state fermentation, the microbial community in cigar tobacco leaves relies on the uncontrolled external migration of microorganisms in the natural environment, which stems from every stage of the production process (Figure 1).

When cigar tobacco leaves grow in the field, microorganisms in the rhizosphere, soil and air gather and adsorb inside and on the surface of the leaves, which is the primary source of the microbial community of cigar tobacco leaves (Yan et al., 2020). The rhizosphere soil of tobacco is a key source of nicotine-degrading microorganisms, as nicotine secreted by tobacco roots into the soil forms a specific nicotine-rich microecological niche, which promotes the colonization and reproduction of nicotine-degrading microorganisms (Deng et al., 2025). The phyllosphere microorganisms of tobacco leaves are mainly from the air and rainwater, and their composition is affected by the planting environment, pesticide application and fertilization measures (Liu et al, 2022b). When cigar tobacco leaves are transferred to the drying room and fermentation room, the microorganisms which inhabit those spaces and exist on the equipment (e.g., wooden barrels, stacking racks) are the secondary source of the microbial community in the leaves. The indigenous microorganisms in the processing environment adapt to the local temperature, humidity and tobacco substrate characteristics, and can quickly colonize and multiply on the tobacco leaves, thus affecting the composition and structure of the microbial community in the subsequent fermentation process.

Quantifying the contribution of potential environmental microbial sources to a specific microbial community not only aids in understanding how microbial communities are formed but also has far-reaching applications in pollution control and microbial community regulation (Wang et al, 2023a; Wang et al, 2023b). At present, microbial source tracking technology based on high-throughput sequencing and multivariate statistical analysis has been used to analyze the contribution rate of different sources to the microbial community of cigar tobacco leaves, which provides a theoretical basis for regulating the microbial community by controlling the source microorganisms.

## 4 Abiotic factors affecting the assembly of microbial communities in cigar tobacco leaves

**FIGURE 2:**
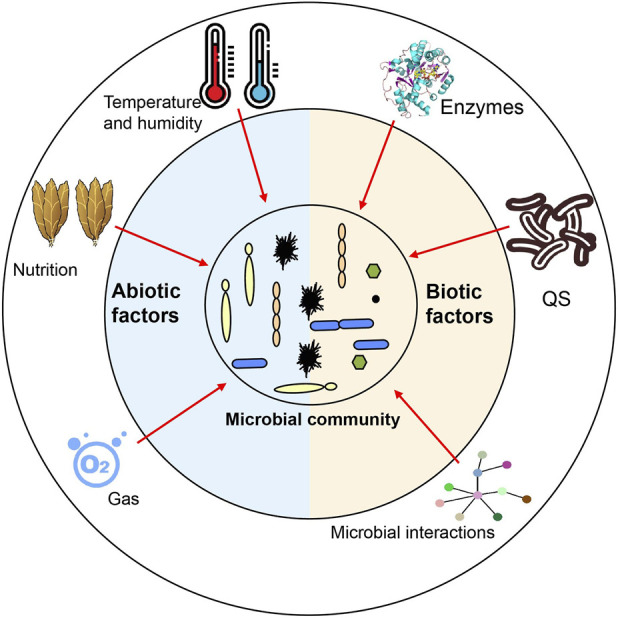
The abiotic and biotic factors affecting the fermentation of cigar tobacco leaves. Abiotic factors include temperature, humidity, oxygen, and nutritional composition. Biotic factors encompass enzymes, Quorum sensing (QS), and microbial interactions.

Once microorganisms migrate to and colonize cigar tobacco leaves, abiotic and biological factors play a decisive role in the assembly of microbial communities (Figure 2). Abiotic conditions can either prevent or promote the establishment of newly arrived microbial communities and can also change the interactions and competitive outcomes among microorganisms, thus indirectly shaping microbial communities. The abiotic factors affecting the microbial community assembly of cigar tobacco leaves mainly include agricultural management measures in the growth stage, and temperature, humidity, oxygen, nutritional composition in the fermentation stage.

### 4.1 Agricultural management measures

During the growth stage of tobacco plants, the first abiotic factor is driven by the activities of plants, animals, or humans (Liu et al, 2021c). Tobacco cultivation commonly involves the application of a large amount of compound fertilizer and farmyard manure. Yan et al. used linear discriminant analysis to identify 40 key phylotypes in response to fertilizers. These phylotypes encompass plant growth-promoting bacteria such as the genera *Sphingomonas*, *Pseudomonas*, *Sphingobium*, *Haliangium* and Rhizobiaceae and *Micrococcus*, which are potentially associated with the quality of the soil and tobacco plants (Yan et al., 2020). In addition, various physical or chemical insecticides are used during the growth process of tobacco plants. Chemical insecticides/fungicides used in tobacco cultivation can reduce the diversity of the indigenous microbial community (especially the phyllosphere microbial community) and inhibit the activity of key functional microorganisms; organic fertilizers can mitigate this impact and promote the growth of beneficial microorganisms (Li, 2022). These activities play an important role in shaping the local microbial species pool that can interact with fermentation.

### 4.2 Temperature and humidity

When cigar tobacco leaves are placed in a controllable fermentation environment, environmental factors, such as temperature, humidity, and flowing air, serve as the second abiotic factor in shaping the microbial community (Ren et al., 2023). Fermentation temperature and humidity comprise two distinct aspects: environmental temperature and humidity, and the temperature and humidity within the stack of cigar tobacco leaves. The initial temperature of the tobacco stack is associated with the ambient temperature. As fermentation proceeds, the tobacco stack gradually warms up under the metabolic activities of the microbial community. Due to temperature differences in different parts of the tobacco stack and the varying temperature adaptability of microorganisms, the spatiotemporal composition and abundance of microbial communities across different layers within the tobacco stack gradually become differentiated. The middle layer of the tobacco stack exhibits the highest niche diversity, the most frequent interspecific interactions, and the highest nutrient utilization rate.

A temperature gradient ranging from 20 °C to 60 °C has been observed to influence the microbial metabolism of *Bacillus*, *Staphylococcus*, and *Aspergillus*, significantly affecting the accumulation of volatile compounds (Jia et al, 2025b). For example, high temperature (45 °C–60 °C) promotes the growth of thermotolerant microorganisms such as *Staphylococcus* and *Bacillus*, and accelerates the degradation of macromolecular substances; moderate temperature (30 °C–40 °C) is conducive to the growth of yeast and *Aspergillus*, and promotes the synthesis of aroma substances such as esters and terpenoids (Zhang et al, 2024b). When the temperature of the middle layer of the tobacco stack approaches the preset temperature range (45 °C–60 °C), the stack needs to be flipped. Flipping the stack means hanging the inner and outer positions as well as the upper and lower positions of the tobacco stack, which ensures uniform and consistent fermentation of the cigar tobacco leaves, eliminates the harmful odors generated by tobacco fermentation and supplement fresh oxygen for aerobic microorganisms.

The moisture content of cigar tobacco leaves has a direct impact on texture, physiology, microbial activities, and enzyme action (Gao et al., 2025; Zhao et al., 2024). When the moisture content is excessive, it reduces the porosity of the substrate and the transportation of oxygen, resulting in the accumulation of water and hindered microbial aeration. Additionally, excessive moisture destroys the balance of the bacterial and fungal community (Zhou et al., 2024), leading to mildew and decay of cigar tobacco leaves because filamentous fungi can overcome oxygen diffusion limitations by forming aerial hyphae (Zhang et al., 2026). However, insufficient moisture impedes nutrient uptake, hinders microbial growth and enzyme production, affecting the fermentation effect of cigar tobacco leaves (Baker et al., 2019). The optimal moisture content for fermentation is 25%–40% (agricultural fermentation) and 20%–30% (industrial fermentation), which can maintain the normal metabolic activity of microorganisms and the smooth progress of enzymatic reactions (Zhang et al, 2024b).

### 4.3 Oxygen

Oxygen concentration is an important abiotic factor regulating the microbial community assembly of cigar tobacco leaves, which can change the fermentation quality by regulating the succession of aerobic and anaerobic microbial communities. Under aerobic conditions, *Cyanobacteria*, *Pseudomonas* and *Aspergillus* are the dominant microorganisms, which are mainly responsible for the degradation of macromolecular substances and the synthesis of aerobic metabolites (e.g., terpenoids, aldehydes). Under oxygen-limited conditions, *Staphylococcus* and *Corynebacterium* are the dominant microorganisms, which promote the formation of anaerobic metabolites (e.g., alcohols, esters) (Yang et al., 2024). In solid-state fermentation, respiratory intensity (oxygen uptake rate and/or carbon dioxide production rate) serves as a reliable indicator for estimating microbial growth and fermentation degrees, which can be used to monitor the fermentation state and adjust the oxygen supply in real time (Oostra et al., 2001).

The oxygen supply of the tobacco stack is mainly adjusted by flipping the stack and controlling the stacking density. Frequent flipping can increase the oxygen content in the tobacco stack and promote the growth of aerobic microorganisms; high stacking density can reduce the oxygen supply and create an oxygen-limited environment for anaerobic microbial metabolism (Zhang et al., 2023a). Different fermentation stages have different oxygen requirements: the agricultural fermentation stage needs sufficient oxygen to promote the degradation of macromolecular substances (Yang et al., 2024); the industrial fermentation stage can appropriately reduce the oxygen supply to promote the synthesis of complex aroma substances (Zhang et al, 2024b).

### 4.4 Nutritional composition

The nutritional composition in cigar tobacco leaves is one of the most important abiotic factors affecting the composition of microbial communities. At different fermentation stages, these are significant variations in the types of carbon sources and nitrogen sources, which is the primary cause of the dynamic succession of the microbial community during the fermentation process of cigar tobacco leaves. Fermentation continuously reshapes the microbial community in cigar tobacco leaves, which in turn affects the fermentation results and the composition of nutritional substances.

In the agricultural fermentation stage, the carbon content in cigar tobacco leaves is approximately 5%–10%, and the content of easily metabolized carbohydrates (e.g., glucose, fructose) is high, which is sufficient to support the growth of most microorganisms. Reducing sugars promote the growth of fast-growing microorganism, such as *Pseudomonas*, yeast, which are the main force for the initial degradation of macromolecules (Zhang et al., 2023a). In the industrial fermentation stage, the content of most easily metabolized carbohydrates declines, and the proportion of slowly degradable carbon sources (e.g., cellulose, hemicellulose) increases, intensifying microbial competition. The change in resource availability is the primary cause of the dynamic succession of the microbial community during the fermentation process of cigar tobacco leaves. At this time, microorganisms with cellulolytic and hemicellulolytic abilities (e.g., *Aspergillus*, *Bacillus*) become the dominant groups, which continue to degrade macromolecular substances and provide nutrients for other microorganisms (Chen et al., 2025).

Nitrogen sources in cigar tobacco leaves include protein, amino acid, nicotine and other nitrogen-containing compounds, among which nicotine is a unique nitrogen source and carbon source in cigar tobacco leaves, and is the core nutritional factor driving the assembly of nicotine-degrading microbial communities (Li et al., 2021). These microorganisms are widely distributed in the tobacco ecosystem and play an important regulatory role in cigar tobacco leaf fermentation by degrading nicotine, which is a key factor affecting the irritation of tobacco leaves. Nicotine-degrading microorganisms degrade nicotine into non-irritating small molecules (e.g., pyrrolidine, organic acids, carbon dioxide and water) through pyridine, pyrrolidine, and a variation of the pyridine and pyrrolidine pathways in bacteria, as well as demethylation pathway in fungi (Meng et al., 2010; Zhang et al, 2022b). In addition, the degradation products of nicotine can be used as carbon and nitrogen sources by other microorganisms, promoting the growth and metabolism of other functional microorganisms (e.g., aroma-synthesizing fungi) and the synthesis of aroma substances, thus indirectly enhancing the flavor quality of cigar tobacco leaves. At present, the application of nicotine-degrading microorganisms in the tobacco industry is mainly focused on tobacco fermentation and tobacco waste treatment (Li et al, 2024b). In cigar fermentation, nicotine-degrading microorganisms are used as functional inoculants to reduce the irritation of tobacco leaves and improve the flavor quality (Zhao et al., 2012); in tobacco waste treatment, nicotine-degrading microorganisms are widely used to degrade nicotine in tobacco waste to reduce environmental pollution (Wang et al, 2023c). However, industrial use is limited by low environmental tolerance, unclear microbial interactions, and slow degradation. Future directions include improving nicotine-degrading microorganism performance via metabolic engineering, constructing SynComs, and developing biopesticides and fertilizers for sustainable tobacco production. Amino acids in cigar tobacco leaves not only provide nitrogen sources for microbial growth, but also participate in the Maillard reaction under the action of microorganisms to form aromatic compounds such as melanoidins and aldehydes (Chen et al., 2025; Si et al., 2023).

## 5 Biotic factors affecting the assembly of microbial communities in cigar tobacco leaves

When the microorganisms migrating into cigar tobacco leaves cannot be filtered out by abiotic factors, biological factors play a decisive role in determining the dominant microorganisms in cigar tobacco leaves (Figure 2). Nevertheless, compared with abiotic selection, it is significantly more challenging to explore and describe the influence of biotic factors on the microbial community. With the application of multi-omics technology to study the microbiome in cigar tobacco leaves, microbial interactions are increasingly regarded as important driving factors for microbial community assembly, which impacts the quality and safety of fermentation. The microbial interactions can be mainly categorized into two types: direct interactions and indirect interactions (Liu et al., 2020).

### 5.1 Direct microbial interactions

Direct interaction refers to the situation where microorganisms directly inhibit or promote the growth and metabolism of neighboring microorganisms through metabolites or other substances they produce (Pierce and Dutton, 2022). The main forms of direct interaction include mutualism, commensalism, competition and antagonism. Mutualism means that two or more microorganisms promote each other’s growth and metabolism, such as the synergy between *Staphylococcus* and *Bacillus*, their complementary enzymatic profiles facilitating the more efficient decomposition of a wider range of compounds (Yao et al., 2025). Commensalism means that one microorganism benefits from the growth and metabolism of another microorganism without affecting the latter. Competition is the most common direct interaction in the microbial community of cigar tobacco leaves, which mainly occurs for nutrients, ecological niches and oxygen. For example, *Acinetobacter* and *Aquabacterium* compete for carbon sources and oxygen in the fermentation process (Zheng et al., 2022b). Antagonism means that one microorganism produces antimicrobial substances (e.g., bacteriocin, antibiotics, volatile organic compounds) to inhibit or kill another microorganism. For example, *Staphylococcus* and *Bacillus* produce bacteriocins to inhibit the growth of other bacteria and fungi (Yao et al., 2025). Direct interactions are often specific and can only affect some microbiotas, which is an important factor maintaining the diversity and stability of the microbial community (Nawaz et al., 2022).

### 5.2 Indirect microbial interactions (substrate mediated interactions)

Indirect interactions (also known as substrate mediated interactions) are the ways in which microorganisms indirectly affect the growth of neighboring microorganisms (Schmidt et al., 2015). In this case, microorganisms do not directly secrete metabolites to affect the growth of other microorganisms. Instead, they produce certain chemical substances or alter the environment in a specific growth mode, thereby changing the growth of other microorganisms. A classic example of substrate mediated interactions in the fermentation of cigar tobacco leaves is the secretion of extracellular enzymes (Ma et al., 2024). These enzymes break down macromolecular substances (cellulose, protein, lipid) and release nutrients (glucose, amino acids, fatty acids) accessible to other microorganisms, which is the basis for the coexistence of different microbial groups in the tobacco leaf substrate.

Small molecules, such as volatile organic compounds (VOCs) produced by microorganisms during fermentation serve as the main pathway for substrate mediated interactions, affecting the physiological characteristics of interacting members (Zhang et al, 2022a). Microorganisms produce a variety of VOCs (e.g., alcohols, aldehydes, esters, terpenoids) during fermentation, which can regulate the spore germination, growth and metabolism of other microorganisms, and even affect the expression of functional genes (e.g., enzyme genes, aroma synthesis genes). For example, the VOCs produced by *Aspergillus* can promote the synthesis of esters by yeast; the VOCs produced by yeast can inhibit the spore germination of harmful fungi. Substrate mediated indirect interactions are usually more extensive and can affect a wide range of microbial communities, which play an important role in the succession of microbial community and the formation of fermentation flavor.

### 5.3 Quorum sensing and its regulatory role

Quorum sensing (QS) is a cell-cell communication mechanism by which microorganisms sense the population density and regulate gene expression and metabolic activity in a population density-dependent manner (Abisado et al., 2018; Zeng et al., 2023). The substances that mediate quorum sensing are commonly known as quorum sensing factors (QS), which are secreted by microorganisms and accumulate in the environment with the increase of population density. To date, a variety of quorum sensing effector molecules have been discovered. Based on the target and molecular composition, they can be divided into four types, including acyl homoserine lactone (AHL) effector molecules of Gram-negative bacteria, autoinducible peptides (AIP) of Gram-positive bacteria, autoinducer class II and other effector molecules (Green et al., 2023; Liu et al, 2022a). Moreover, some quinolones, some esters and fatty acids can also serve as quorum sensing signals in cigar tobacco leaf fermentation (Zeng et al., 2023).

In the fermentation of cigar tobacco leaves, several indigenous strains involved in or relying on quorum sensing systems have been reported to play functional roles. For example, *Pseudomonas* produce AHL-type QS molecules that regulate the secretion of cellulases and proteases, which are critical for macromolecular degradation and aroma precursor formation (Lv et al., 2013). *Staphylococcus* use AIP-mediated quorum sensing to modulate biofilm formation and metabolic product synthesis, affecting aroma compound accumulation and microbial colonization (Tal-Gan et al., 2013). Additionally, *Aspergillus* secrete fatty acid-derived QS signals that coordinate the expression of lipase genes, promoting lipid degradation and volatile ester formation (Qi et al., 2022). By regulating the QS system of microbial communities, the metabolic activity and interaction of microorganisms can be controlled, so as to realize the precise regulation of cigar tobacco leaf fermentation.

### 5.4 Analysis methods of microbial interactions

Currently, the main method for analyzing potential microbial interactions in cigar tobacco leaf fermentation primarily relies on Spearman correlation and co-occurrence network analysis of amplicon or metagenomic sequencing data (Fang et al., 2024). It has been found that *Staphylococcus* and *Corynebacterium* generally showed negative correlations with other bacteria, while *Curtobacterium* mainly had positive correlations with other bacteria. Notably, bacteria were negatively correlated with fungi, and bacterial interactions seem more robust than those within fungal communities. This method can quickly identify the potential interaction relationships between microbial species and construct the microbial co-occurrence network, which provides a preliminary understanding of the microbial community structure.

In addition, genome-scale metabolic networks based on omics data (metagenomics, metabolomics, transcriptomics) are often used to explore the metabolic interactions between microorganisms (Gong et al., 2024). This method can analyze the metabolic complementarity and competition between microorganisms at the molecular level, and identify the key metabolic pathways and metabolites involved in microbial interactions. Another more in-depth approach is to measure the impact of individual microorganisms on the community by single strain addition experiments (Hsu et al., 2019). By adding individual species each time, the final impact of a species on the rest of the community can be measured, and the direct and indirect interactions between microorganisms can be identified (Hu et al., 2022). In our previous study, it was found that inoculated *Acinetobacter* directly affected the growth of *Aquabacterium* and *Bacillus*, and then indirectly affected other microbiotas, such as *Staphylococcus*, *Aerococcus*, *Lutispora*, and *Zoogloea*. *Acinetobacter* showed a negative interaction with *Aquabacterium* and a positive correlation with *Bacillus* (Zheng et al., 2022a). This method helps to quickly capture the consequences of direct and indirect interactions and identify highly influential or interacting species that may play an important role in shaping the entire microbial community.

## 6 Regulation of microbial community to improve fermentation quality of cigar tobacco leaves

**FIGURE 3:**
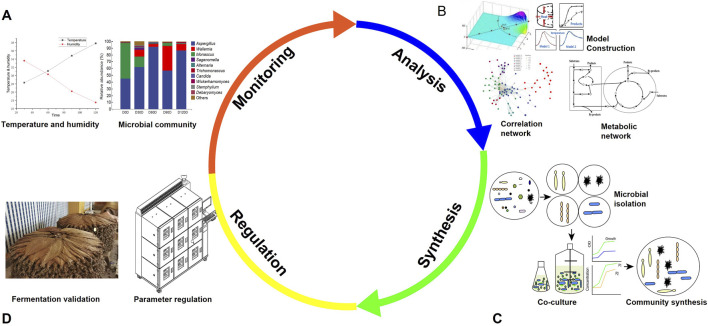
Schematic overview of the fermentation regulation system for cigar tobacco leaves. **(A)** Real-time monitoring of fermentation: Real-time tracking of fermentation parameters (temperature and humidity) and dynamic changes in microbial community composition during the fermentation process. **(B)** Big data analysis: Constructing models through multi-dimensional data analysis to decode the complex relationships between environmental factors, microbial communities, and fermentation products. **(C)** Synthetic microbial community: Design and construction of synthetic microbial communities through isolation, cultivation, co-cultivation. **(D)** Fermentation regulation: The synthetic microbial community and big data analysis results were experimented in an intelligent fermentation system, achieving precise adjustment and optimization of fermentation parameters, and validated in fermentation production.

Once various abiotic and biotic factors that influence microbial community assembly have been identified, we can precisely control these factors to achieve the desired fermentation outcomes (Figure 3). The regulation strategies of microbial community in cigar tobacco leaf fermentation mainly include environmental condition regulation, artificial intelligence-based intelligent regulation, and synthetic microbial community regulation. These strategies can be used alone or in combination to realize the precise and efficient regulation of microbial community, and improve the fermentation quality and stability of cigar tobacco leaves.

### 6.1 Environmental condition regulation

Adjusting the environmental conditions of fermentation to regulate the microbial community is the simplest and most widely used method for cigar producers. Currently, a real-time monitoring and remote transmission system has been employed to monitor the fermentation environment (temperature, humidity, oxygen content, stack temperature). This allows us to adjust the fermentation conditions promptly according to the fermentation process of cigar tobacco leaves. By reasonably controlling temperature and humidity, we can promote the growth of beneficial bacteria and fungi (e.g., *Aspergillus*, *Staphylococcus*) while suppressing the growth of harmful molds (e.g., *Penicillium*, *Fusarium*). For example, controlling the fermentation temperature at 30 °C–40 °C and moisture content at 25%–30% can promote the growth of aroma-synthesizing fungi and yeast; appropriately increasing the temperature to 45 °C–50 °C can inhibit the growth of harmful molds and accelerate the degradation of macromolecules (Jia et al, 2025b; Ren et al., 2023).

In addition, molecular biology techniques, such as quantitative real-time polymerase chain reaction (qPCR) and next-generation sequencing, are used to monitor the composition and changes of the microbial community during the fermentation process (Jia et al, 2025a; Taylor et al., 2019). Regularly analyzing the microbial community structure allows for the timely detection of any abnormal changes. For example, if the proportion of a certain harmful mold suddenly rises, corresponding measures like adjusting the environmental conditions (e.g., reducing moisture, increasing flipping frequency) or adding appropriate inhibitors can be taken. Figure 3A shows the core indicator microorganisms in each stage of fermentation, which are the key monitoring objects of the real-time monitoring system, and their community structure changes can directly reflect the fermentation state of cigar tobacco leaves.

### 6.2 Artificial intelligence-based intelligent regulation

With the rapid development of artificial intelligence technologies, such as artificial sensors, spectroscopy, computer vision, digital image processing, machine learning and big data analysis, it has become feasible to quantify temperature and humidity changes, as well as succession patterns of microbial communities during the fermentation of cigar tobacco leaves (Liu et al, 2021b; Yang et al., 2023). The intelligent regulation system of cigar tobacco leaf fermentation based on artificial intelligence mainly includes four parts: real-time monitoring, big data analysis, quality prediction and automatic control (Figures 3B,D).

Computer vision technology can be used to monitor the physical appearance of cigar tobacco leaves (color, texture, shape) (Yin et al., 2024). It can detect any abnormal color changes (e.g., mildew, discoloration) or texture alterations that might indicate problems in the fermentation process, and realize the non-destructive monitoring of fermentation state. Spectroscopy technology (near-infrared spectroscopy, Raman spectroscopy) can quickly detect the content of chemical components (nicotine, reducing sugar, aroma substances) in tobacco leaves without destroying the sample, and reflect the fermentation progress and flavor formation state (Yan et al., 2026). Big data analysis can further help in comparing different batches of fermentation, enabling producers to understand the similarities and differences in the fermentation process and outcomes (Ammar et al., 2022). Consequently, more targeted adjustments can be made in subsequent fermentations.

Machine learning algorithms can identify key factors closely related to high-quality fermentation by analyzing data collected from sensors, spectroscopic devices and high-throughput sequencing, such as specific temperature ranges, humidity levels, oxygen concentration and the presence of certain types of microorganisms. These key factors can be used to construct a fermentation quality prediction model, which can accurately predict the quality of cigar tobacco leaf fermentation and provide scientific guidance for optimizing the fermentation process (Xing et al., 2025a). Additionally, artificial intelligence-based systems can be integrated with automated control systems in fermentation facilities (Figure 3D). This means that when the system detects that a certain factor is deviating from the optimal range, it can automatically adjust environmental parameters such as temperature, humidity, flipping frequency and oxygen supply to ensure that the fermentation process stays on course.

The application of artificial intelligence technology can contribute to the standardization and industrialization of cigar production. Clear data on the fermentation process make it easier to establish industry-wide standards, which is beneficial for both producers and consumers. Producers can better meet market demands, and consumers can anticipate a more reliable and enjoyable cigar-smoking experience. However, the application of artificial intelligence technology in this field also encounters challenges. For example, the high complexity of microbial communities in cigar tobacco leaves may pose difficulties in accurately identifying and analyzing relevant data; the cost of implementing artificial intelligence-related technologies may be relatively high, which could limit their large-scale application in some cigar-producing areas. In the future, with the development of artificial intelligence technology and the reduction of application cost, the intelligent regulation system will become the core technology of cigar tobacco leaf fermentation industrialization.

### 6.3 Synthetic microbial community regulation

The use of microbial interactions to regulate the community is of great significance in the fermentation process of cigar tobacco leaves (Iven et al., 2022). For example, enhancing the symbiotic relationship among microorganisms enables them to cooperate to complete the decomposition of complex substances in cigar tobacco leaves; adjusting the competitive relationship between microorganisms allows them to gain a dominant position in the competition with harmful microorganisms. The most common method is to add specific strains of beneficial bacteria and fungi as inoculants to regulate the microbial community. For example, certain lactic acid bacteria can produce lactic acid during fermentation, lowering the pH of cigar tobacco leaves, inhibiting the growth of harmful microorganisms and promoting the breakdown of proteins and starches in the tobacco leaves, thereby improving the flavor quality (Zhang et al, 2025b). Certain *Cyberlindnera fabianii* can also be used as inoculants, which are involved in the decomposition of complex organic substances and the formation of flavor precursors (Guo et al., 2024a).

Instead of using a single strain, the application of a synthetic microbial community composed of multiple beneficial microorganisms can have a more extensive and comprehensive effect (Figure 3C) (Men et al., 2026). Within this synthetic microbial community, different microorganisms can engage in synergistic interactions. They can complement each other in aspects such as nutrient utilization, metabolite production, and the inhibition of harmful microorganisms (Mehlferber et al., 2024). Through these interactions, the overall fermentation quality is enhanced, and the stability of fermentation process is improved. The preparation of SynComs usually adopts top-down and bottom-up approaches (Gao et al., 2023; Jin et al., 2024), and the combination of the two approaches can fully leverage their advantages and compensate for each other’s shortcomings.

The bottom-up approach starts from a single microbial strain and gradually builds a complex microbial community according to pre-designed functions and interactions (Du et al., 2023). This method is based on an in-depth understanding of the individual characteristics and interaction mechanism of microorganisms, and realizes the precise control of community structure and function by accurately regulating the species, quantity and culture conditions of microorganisms. The bottom-up approach has high controllability and reproducibility, and can accurately study the interaction mechanisms between microorganisms, providing powerful tools for a deeper understanding of the functions of microbial communities. In addition, this method can design and construct microbial communities with specific functions according to actual needs, and has strong application specificity. For example, constructing a synthetic microbial community composed of nicotine-degrading microorganisms (*Pseudomonas sp.*), starch-degrading bacteria (*Bacillus sp.*) and aroma-synthesizing fungi (*Aspergillus sp.*) can simultaneously realize nicotine degradation, macromolecule decomposition and aroma synthesis (Men et al., 2026). However, this method requires a deep understanding of the characteristics and interactions of individual microorganisms, but the current understanding of the complex interactions between microorganisms in cigar fermentation is not comprehensive enough, which may result in the constructed community not achieving the expected function. In addition, as the complexity of the community increases, the difficulty of construction and regulation will also significantly increase.

The top-down approach is to simplify, transform and optimize the existing complex microbial community in the natural environment to obtain a synthetic microbial community with specific functions (Gilmore et al., 2019). The top-down approach fully utilizes the diversity and functional potential of natural microbial communities, enabling the rapid acquisition of SynComs with complex functions, which is suitable for solving some practical application problems (Jiang et al., 2024). Moreover, this method relies relatively less on the interactions between microorganisms and places more emphasis on achieving the overall functionality of the community. Due to the complexity of natural microbial communities, it is difficult to precisely regulate and optimize them, making it difficult to achieve precise control over community structure and function. At the same time, this method may be subject to interference from complex interactions between microorganisms in the natural environment, resulting in unstable or unsustainable functioning of the modified community.

The combination of bottom-up and top-down synthetic microbial community methods can provide new ideas and strategies for building more stable, efficient, and functionally diverse SynComs for cigar fermentation. This integrated approach could involve initially using the bottom-up method to design a synthetic microbial community with a basic framework of desired functions and then using the top-down method to fine-tune the community under specific cigar fermentation environmental conditions (e.g., adding indigenous microorganisms adapted to local tobacco leaves) to ensure its stability and optimal performance. At present, the research on synthetic microbial community in cigar fermentation is still in the lab-scale stage, and most studies are based on tobacco/cigar fermentation-specific research. The main challenges are the low stability of synthetic microbial community in industrial fermentation environment, the unclear interaction between synthetic microbial community and indigenous microbial communities, and the high cost of community construction. In the future, with the in-depth study of microbial interactions and the development of synthetic microbiology technology, synthetic microbial community will become an important technology for the precise regulation of cigar tobacco leaf fermentation.

## 7 Summarization and prospects

In conclusion, this review systematically analyzes the sources, ecology, and evolution of microbial communities in cigar tobacco leaves, along with their impact on microbial community assembly. Understanding the mechanism of microbial community assembly in cigar tobacco leaves allows us to utilize abiotic and biological selection pressures to regulate and optimize the fermentation of cigar tobacco leaves. Moreover, the integration of artificial intelligence technology can assist us in better regulating the fermentation process of cigar tobacco leaves and controlling their fermentation quality.

## References

[B1] Abisado R. G. Benomar S. Klaus J. R. Dandekar A. A. Chandler J. R. (2018). Bacterial quorum sensing and microbial community interactions. mBio 9 (3) 10.1128/mBio.02331-17 29789364 PMC5964356

[B2] Ammar K. A. Kheir A. M. S. Manikas I. (2022). Agricultural big data and methods and models for food security analysis-a mini-review. PeerJ 10, e13674. 10.7717/peerj.13674 35789661 PMC9250308

[B3] Baker P. W. Charlton A. Hale M. D. C. (2019). Fibre degradation of wheat straw by Pleurotus erygnii under low moisture conditions during solid-state fermentation. Lett. Appl. Microbiol. 68 (2), 182–187. 10.1111/lam.13104 30516831

[B4] Banožić M. Jokić S. Ačkar Đ. Blažić M. Šubarić D. (2020). Carbohydrates—key players in tobacco aroma formation and quality determination. Molecules 25 (7), 1734. 10.3390/molecules25071734 32283792 PMC7181196

[B5] Blanchard L. Tominaga T. Dubourdieu D. (2001). Formation of furfurylthiol exhibiting a strong coffee aroma during oak barrel fermentation from furfural released by toasted staves. J. Agric. Food Chem. 49 (10), 4833–4835. 10.1021/jf010539w 11600030

[B6] Chai L. J. Qian W. Zhong X.-Z. Zhang X. J. Lu Z. M. Zhang S. Y. (2021). Mining the factors driving the evolution of the pit mud microbiome under the impact of long-term production of strong-flavor baijiu. Appl. Environ. Microbiol. 87 (17), e0088521. 10.1128/AEM.00885-21 34160281 PMC8357292

[B7] Chen S. Zhu F. Zhang S. Wang S. Shen Y. Zhang M. (2025). Integrated analysis of proteome and metabolome reveals the basis of amino acid metabolism in cigar artificial fermentation. Appl. Biochem. Biotechnol. 197 (8), 5308–5326. 10.1007/s12010-025-05275-4 40434605

[B8] Deng M. Basak P. Zhang Y. Song J. Suo H. (2025). An update in recent research on nicotine contamination and nicotine-degrading microorganisms. Toxicon 254, 108209. 10.1016/j.toxicon.2024.108209 39662531

[B9] Du R. Jiang J. Qu G. Wu Q. Xu Y. (2023). Directionally controlling flavor compound profile based on the structure of synthetic microbial community in Chinese liquor fermentation. Food Microbiol. 114, 104305. 10.1016/j.fm.2023.104305 37290868

[B10] Fang X. Qin Y. Liu T. Guo S. Wu C. Zhang R. (2024). Roles of cigar microbes in flavor formation during roasted-rice leachate fermentation. Appl. Microbiol. Biotechnol. 108 (1), 457. 10.1007/s00253-024-13289-x 39222255 PMC11368999

[B11] Gao H. Jiang W. Zhang W. Jiang M. Xin F. (2023). Customized spatial niches for synthetic microbial consortia. Trends Biotechnol. 41 (12), 1463–1466. 10.1016/j.tibtech.2023.05.003 37270330

[B12] Gao Y. Wang Y. Hou B. Zhang G. Jiang C. Fang S. (2025). Diversity of microbial communities in cigar filler leaves with different initial water contents analyzed based on high-throughput sequencing technology. Front. Microbiol. 16, 1508866. 10.3389/fmicb.2025.1508866 39990154 PMC11845121

[B13] Gilmore S. P. Lankiewicz T. S. Wilken S. E. Brown J. L. Sexton J. A. Henske J. K. (2019). Top-down enrichment guides in formation of synthetic microbial consortia for biomass degradation. ACS Synth. Biol. 8 (9), 2174–2185. 10.1021/acssynbio.9b00271 31461261

[B14] Gong X. W. Yang J. K. Duan Y. Q. Dong J. Y. Zhe W. Wang L. (2009). Isolation and characterization of Rhodococcus sp. Y22 and its potential application to tobacco processing. Res. Microbiol. 160 (3), 200–204. 10.1016/j.resmic.2009.02.004 19298855

[B15] Gong Z. Chen J. Jiao X. Gong H. Pan D. Liu L. (2024). Genome-scale metabolic network models for industrial microorganisms metabolic engineering: current advances and future prospects. Biotechnol. Adv. 72, 108319. 10.1016/j.biotechadv.2024.108319 38280495

[B16] Green M. J. Murray E. J. Williams P. Ghaemmaghami A. M. Aylott J. W. Williams P. M. (2023). Modelled-microgravity reduces virulence factor production in Staphylococcus aureus through downregulation of agr-dependent quorum sensing. Int. J. Mol. Sci. 24 (21), 15997. 10.3390/ijms242115997 37958979 PMC10648752

[B17] Guo S. Li Y. Yang Z. Zhang Q. Li P. Jiang Z. (2024a). Isolation and evaluation of Cyberlindnera fabianii strains to improve cigar tobacco leaves fermentation effect. Front. Microbiol. 15, 1492042. 10.3389/fmicb.2024.1492042 39720475 PMC11666510

[B18] Guo S. Li Y. Zhu B. Zhang Q. Yang Z. Jia Y. (2024b). Introducing CCD1 into isolated Rhodotorula strain enhances flavor production and improves cigar fermentation. Front. Bioeng. Biotechnol. 12, 1510075. 10.3389/fbioe.2024.1510075 39691208 PMC11650503

[B19] Hsu R. H. Clark R. L. Tan J. W. Ahn J. C. Gupta S. Romero P. A. (2019). Microbial interaction network inference in microfluidic droplets. Cell Syst. 9 (3), 229–242.e4. 10.1016/j.cels.2019.06.008 31494089 PMC6763379

[B20] Hu W. R. Cai W. Zheng Z. J. Liu Y. F. Luo C. Xue F. (2022). Study on the chemical compositions and microbial communities of cigar tobacco leaves fermented with exogenous additive. Sci. Rep. 12 (1), 19182. 10.1038/s41598-022-23419-y 36357535 PMC9649726

[B21] Iven H. Walker T. W. N. Anthony M. (2022). Biotic interactions in soil are underestimated drivers of microbial carbon use efficiency. Curr. Microbiol. 80 (1), 13. 10.1007/s00284-022-02979-2 36459292 PMC9718865

[B22] Jia Y. Liu Y. Hu W. Cai W. Zheng Z. Luo C. (2023). Development of Candida autochthonous starter for cigar fermentation via dissecting the microbiome. Front. Microbiol. 14, 1138877. 10.3389/fmicb.2023.1138877 36910204 PMC9998997

[B23] Jia K. Shi J. Bai L. Wang X. Wang Y. Li X. (2025a). Integrated transcriptomic and metabolomic analysis of flavonoid biosynthesis in cigar tobacco leaves under variable nitrogen regimes. Front. Plant Sci. 16, 1589215. 10.3389/fpls.2025.1589215 40630733 PMC12235917

[B24] Jia Y. Guo S. Hu W. Zhang Q. Wang Y. Zhang Z. (2025b). Effects of different fermentation temperatures on microbiomes of cigar tobacco leaves. Front. Bioeng. Biotechnol. 25, 13. 10.3389/fbioe.2025.1550383 40070551 PMC11893599

[B25] Jiang X. Peng Z. Zhang J. (2024). Starting with screening strains to construct synthetic microbial communities (SynComs) for traditional food fermentation. Food Res. Int. 190, 114557. 10.1016/j.foodres.2024.114557 38945561

[B26] Jin R. Song J. Liu C. Lin R. Liang D. Aweya J. J. (2024). Synthetic microbial communities: novel strategies to enhance the quality of traditional fermented foods. Compr. Rev. Food Sci. Food Saf. 23 (4), e13388. 10.1111/1541-4337.13388 38865218

[B27] Jiranek V. (2024). The prospect of superior yeast for winemaking: recent successes through bioprospecting. Curr. Opin. Biotechnol. 90, 103200. 10.1016/j.copbio.2024.103200 39288658

[B28] Li Z. (2022). Modeling pesticide residues in tobacco leaves for improving life cycle inventory analysis of pesticides in the cigarette industry. Sci. Total Environ. 845, 157267. 10.1016/j.scitotenv.2022.157267 35820521

[B29] Li J. Yi F. Chen G. Pan F. Yang Y. Shu M. (2021). Function enhancement of a metabolic module via endogenous promoter replacement for pseudomonas sp. JY-Q to degrade nicotine in tobacco waste treatment. Appl. Biochem. Biotechnol. 193 (9), 2793–2805. 10.1007/s12010-021-03566-0 34061306

[B30] Li W. Yu J. Li H. Yang C. Peng Z. Zhang J. (2024a). The dynamics of microbial community structure and metabolic function in different parts of cigar tobacco leaves during air-curing. Front. Microbiol. 15, 1438566. 10.3389/fmicb.2024.1438566 39726961 PMC11669699

[B31] Li Z. J. Yang D. D. Wei Z. Y. Huang J. Chi Y. Q. Lu Y. X. (2024b). Reduction of nicotine content in tobacco through microbial degradation: research progress and potential applications. Biotechnol. Biofuels Bioprod. 17 (1), 144. 10.1186/s13068-024-02593-3 39695820 PMC11656995

[B32] Liu F. Mao J. Kong W. Hua Q. Feng Y. Bashir R. (2020). Interaction variability shapes succession of synthetic microbial ecosystems. Nat. Commun. 11 (1), 309. 10.1038/s41467-019-13986-6 31949154 PMC6965111

[B33] Liu F. Wu Z. Zhang X. Xi G. Zhao Z. Lai M. (2021a). Microbial community and metabolic function analysis of cigar tobacco leaves during fermentation. MicrobiologyOpen 10 (2), e1171. 10.1002/mbo3.1171 33970539 PMC8483401

[B34] Liu P. R. Lu L. Zhang J. Y. Huo T. T. Liu S. X. Ye Z. W. (2021b). Application of artificial intelligence in medicine: an overview. Curr. Med. Sci. 41 (6), 1105–1115. 10.1007/s11596-021-2474-3 34874486 PMC8648557

[B35] Liu X. Wei Z. Ma Y. Liu J. Liu F. (2021c). Effects of biochar amendment and reduced irrigation on growth, physiology, water-use efficiency and nutrients uptake of tobacco (Nicotiana tabacum L.) on two different soil types. Sci. Total Environ. 770, 144769. 10.1016/j.scitotenv.2020.144769 33736368

[B36] Liu L. Zeng X. Zheng J. Zou Y. Qiu S. Dai Y. (2022a). AHL-mediated quorum sensing to regulate bacterial substance and energy metabolism: a review. Microbiol. Res. 262, 127102. 10.1016/j.micres.2022.127102 35792523

[B37] Liu T. T. Guo S. P. Wu C. D. Zhang R. N. Zhong Q. Shi H. Z. (2022b). Phyllosphere microbial community of cigar tobacco and its corresponding metabolites. Front. Microbiol. 13, 1025881. 10.3389/fmicb.2022.1025881 36439836 PMC9691965

[B38] Liu H. Zhang H. Powell J. Delgado-Baquerizo M. Wang J. Singh B. (2023). Warmer and drier ecosystems select for smaller bacterial genomes in global soils. Imeta 2 (1), e70. 10.1002/imt2.70 38868347 PMC10989973

[B39] Lu Z. M. Wang Z. M. Zhang X. J. Mao J. Shi J. S. Xu Z. H. (2018). Microbial ecology of cereal vinegar fermentation: insights for driving the ecosystem function. Curr. Opin. Biotechnol. 49, 88–93. 10.1016/j.copbio.2017.07.006 28843369

[B40] Lv D. Ma A. Tang X. Bai Z. Qi H. Zhuang G. (2013). Profile of the culturable microbiome capable of producing acyl-homoserine lactone in the tobacco phyllosphere. J. Environ. Sci. (China). 25 (2), 357–366. 10.1016/s1001-0742(12)60027-8 23596957

[B41] Ma L. Wang Y. Wang X. Lü X. (2024). Solid-state fermentation improves tobacco leaves quality via the screened Bacillus subtilis of simultaneously degrading starch and protein ability. Appl. Biochem. Biotechnol. 196 (1), 506–521. 10.1007/s12010-023-04486-x 37148443

[B42] Mai J. Liu A. Li W. Lin L. Sun M. L. Wang K. (2025). Biotechnological production of carotenoids using Yarrowia lipolytica. J. Agric. Food Chem. 73 (12), 7034–7045. 10.1021/acs.jafc.4c11251 40079666

[B43] Maltseva P. Y. Plotnitskaya N. A. Ivshina I. B. (2024). Transformation of terpenoids and steroids using actinomycetes of the genus rhodococcus. Molecules 29 (14), 3378. 10.3390/molecules29143378 39064956 PMC11279926

[B44] Martínez-Martínez V. Baladrón C. Gomez-Gil J. Ruiz-Ruiz G. Navas-Gracia L. M. Aguiar J. M. (2012). Temperature and relative humidity estimation and prediction in the tobacco drying process using artificial neural networks. Sensors (Basel) 12 (10), 14004–14021. 10.3390/s121014004 23202032 PMC3545603

[B45] Mehlferber E. C. Arnault G. Joshi B. Partida-Martinez L. P. Patras K. A. Simonin M. (2024). A cross-systems primer for synthetic microbial communities. Nat. Microbiol. 9 (11), 2765–2773. 10.1038/s41564-024-01827-2 39478083 PMC11660114

[B46] Men K. F. Xuan H. Q. Chen J. R. Li H. L. Chen X. W. Jiao Y. L. (2026). Synthetic microbial communities enhance tobacco quality by driving bacterial community succession and modulating metabolite profiles. Ind. Crop. Prod. 240, 122621. 10.1016/j.indcrop.2025.122621

[B47] Meng X. J. Lu L. L. Gu G. F. Xiao M. (2010). A novel pathway for nicotine degradation by Aspergillus oryzae 112822 isolated from tobacco leaves. Res. Microbiol. 161 (7), 626–633. 10.1016/j.resmic.2010.05.017 20600861

[B48] Moreira R. C. Leonardi G. R. Bicas J. L. (2024). Lipase-mediated alcoholysis for *in situ* production of ester bioaromas in licuri oil for cosmetic applications. J. Biotechnol. 392, 25–33. 10.1016/j.jbiotec.2024.06.010 38876312

[B49] Nawaz M. Z. Subin Sasidharan R. Alghamdi H. A. Dang H. (2022). Understanding interaction patterns within deep-sea microbial communities and their potential applications. Mar. Drugs. 20 (2), 108. 10.3390/md20020108 35200637 PMC8874374

[B50] Oostra J. le Comte E. P. van den Heuvel J. C. Tramper J. Rinzema A. (2001). Intra-particle oxygen diffusion limitation in solid-state fermentation. Biotechnol. Bioeng. 75 (1), 13–24. 10.1002/bit.1159 11536122

[B51] Pei Q. Jiang X. Li Z. Q. Xu H. Xie M. Y. Xiong T. (2025). Study on quality enhancement during cigar tobacco fermentation by staphylococcus nepalensis: insights into microbial community, volatile substances and sensory evaluation. Front. Microbiol. 16, 1526178. 10.3389/fmicb.2025.1526178 40008043 PMC11850395

[B52] Pierce E. C. Dutton R. J. (2022). Putting microbial interactions back into community contexts. Curr. Opin. Microbiol. 65, 56–63. 10.1016/j.mib.2021.10.008 34739927

[B53] Qi J. Xiao X. Ouyang L. Yang C. Zhuang Y. Zhang L. (2022). Enhancement of fatty acid degradation pathway promoted glucoamylase synthesis in Aspergillus niger. Microb. Cell Fact. 21 (1), 238. 10.1186/s12934-022-01966-3 36376878 PMC9664828

[B54] Ren M. Qin Y. Zhang L. Zhao Y. Zhang R. Shi H. (2023). Effects of fermentation chamber temperature on microbes and quality of cigar wrapper tobacco leaves. Appl. Microbiol. Biotechnol. 107 (21), 6469–6485. 10.1007/s00253-023-12750-7 37665370

[B55] Schmidt R. Cordovez V. de Boer W. Raaijmakers J. Garbeva P. (2015). Volatile affairs in microbial interactions. Isme J. 9 (11), 2329–2335. 10.1038/ismej.2015.42 26023873 PMC4611499

[B56] Si H. Zhou K. Zhao T. Cui B. Liu F. Zhao M. (2023). The bacterial succession and its role in flavor compounds formation during the fermentation of cigar tobacco leaves. Bioresour. Bioprocess. 10 (1), 74. 10.1186/s40643-023-00694-9 38647588 PMC10992852

[B57] Song H. S. Whon T. W. Kim J. Lee S. H. Kim J. Y. Kim Y. B. (2020). Microbial niches in raw ingredients determine microbial community assembly during kimchi fermentation. Food Chem. 318, 126481. 10.1016/j.foodchem.2020.126481 32126467

[B58] Song W. Chen X. Yu J. Qiao J. Y. Yang J. P. Chen X. (2024). Effects of Bacillus altitudinis inoculants on cigar tobacco leaf fermentation. Front. Bioeng. Biotechnol. 12, 1417601. 10.3389/fbioe.2024.1417601 39045536 PMC11264575

[B59] Stubbs J. (2010). El Habano and the world it has shaped: cuba, Connecticut, and Indonesia. Cuban Stud. 41, 39–67. 21506307

[B60] Su Y. B. Cui Y. H. Fu K. J. Bu L. D. Sun Y. C. Zhou Q. (2024). Contribution of pectin-degrading bacteria to the quality of cigar fermentation: an analysis based on microbial communities and physicochemical components. Front. Microbiol. 15, 1481158. 10.3389/fmicb.2024.1481158 39611089 PMC11604125

[B61] Tal-Gan Y. Ivancic M. Cornilescu G. Cornilescu C. C. Blackwell H. E. (2013). Structural characterization of native autoinducing peptides and abiotic analogues reveals key features essential for activation and inhibition of an AgrC quorum sensing receptor in Staphylococcus aureus. J. Am. Chem. Soc. 135 (49), 18436–18444. 10.1021/ja407533e 24219181 PMC3887510

[B62] Taylor S. C. Nadeau K. Abbasi M. Lachance C. Nguyen M. Fenrich J. (2019). The ultimate qPCR experiment: producing publication quality, reproducible data the first time. Trends Biotechnol. 37 (7), 761–774. 10.1016/j.tibtech.2018.12.002 30654913

[B63] Usami A. (2025). Development of biocatalysts for high-value-added compounds. Biosci. Biotechnol. Biochem. 89 (4), 496–501. 10.1093/bbb/zbae139 39384613

[B64] Wang M. Peng S. Liu D. Long D. Liu Z. Pu S. (2023a). Characteristics and traceability analysis of microbial assemblage in fine particulate matter from a pig house. Anim. (Basel) 13 (6), 1058. 10.3390/ani13061058 36978598 PMC10044456

[B65] Wang X. W. Wu L. Dai L. Yin X. Zhang T. Weiss S. T. (2023b). Ecological dynamics imposes fundamental challenges in community-based microbial source tracking. Imeta 2 (1), e75. 10.1002/imt2.75 38868341 PMC10989786

[B66] Wang Y. Luo X. Chu P. Shi H. Wang R. Li J. (2023c). Cultivation and application of nicotine-degrading bacteria and environmental functioning in tobacco planting soil. Bioresour. Bioprocess. 10 (1), 10. 10.1186/s40643-023-00630-x 38647817 PMC10992035

[B67] Wang H. Guo D. Zhang M. Wu G. Shi Y. Zhou J. (2024). Correlation study on microbial communities and volatile flavor compounds in cigar tobacco leaves of diverse origins. Appl. Microbiol. Biotechnol. 108 (1), 236. 10.1007/s00253-024-13032-6 38407656 PMC10896874

[B68] Wen L. Cao J. Li W. Guo Y. (2023). Changes in volatile profile and related gene expression during senescence of tobacco leaves. J. Sci. Food Agric. 103 (13), 6540–6552. 10.1002/jsfa.12733 37223951

[B69] Xing Z. Shi Y. Pan Y. Zhang K. Wang Z. Liu B. (2025a). CADFFNet: a dual-branch neural network for non-destructive detection of cigar leaf moisture content during air-curing stage. Front. Plant Sci. 16, 1698427. 10.3389/fpls.2025.1698427 41267935 PMC12627023

[B70] Xing Z. Shi Y. Zhang K. Ding S. Shi X. (2025b). Moisture content prediction of cigar leaves air-curing process based on stacking ensemble learning model. Front. Plant Sci. 16, 1553110. 10.3389/fpls.2025.1553110 40206878 PMC11979227

[B71] Yan S. Zhao J. Ren T. Liu G. (2020). Correlation between soil microbial communities and tobacco aroma in the presence of different fertilizers. Ind. Crop. Prod. 151, 112454. 10.1016/j.indcrop.2020.112454

[B72] Yan R. Li A. Yang W. Lei Y. Qin Y. Song Z. (2026). Composition and ultrastructure changes of leaf cuticle wax during the air-curing process in cigars. Plant Physiol. Biochem. 231, 111074. 10.1016/j.plaphy.2026.111074 41579694

[B73] Yang B. Yang S. Zhu X. Qi M. Li H. Lv Z. (2023). Computer vision technology for monitoring of indoor and outdoor environments and HVAC equipment: a review. Sensors (Basel) 23 (13), 6186. 10.3390/s23136186 37448035 PMC10346506

[B74] Yang J. Xue F. Li D. Chen J. Shi G. Song G. (2024). Oxygen regulation of microbial communities and chemical compounds in cigar tobacco curing. Front. Microbiol. 15, 1425553. 10.3389/fmicb.2024.1425553 39109208 PMC11300322

[B75] Yao L. Zhao Z. Li L. Yu J. Yang J. Yang C. (2025). The impact of Staphylococcus saprophyticus on the fermentation of cigar filler tobacco leaves and the dynamics of microbial community. Front. Bioeng. Biotechnol. 13, 1666879. 10.3389/fbioe.2025.1666879 41064528 PMC12500701

[B76] Yin J. Wang J. Jiang J. Xu J. Zhao L. Hu A. (2024). Quality prediction of air-cured cigar tobacco leaf using region-based neural networks combined with visible and near-infrared hyperspectral imaging. Sci. Rep. 14 (1), 31206. 10.1038/s41598-024-82586-2 39732746 PMC11682218

[B77] Yin Y. M. Song X. R. Cui Y. H. Zhang J. L. Fu K. J. Zhou Q. (2025). Application of starch-degradation bacteria in cigar tobacco leaf fermentation: effects on starch degradation, microbial communities and metabolic pathways. Front. Microbiol. 16, 1632731. 10.3389/fmicb.2025.1632731 40761280 PMC12319561

[B78] Yuan Y. Zhang W. Pang J. Zhou M. Liu J. Zhao J. (2023). Integrated physiological and metabolomic analyses reveal changes during the natural senescence of Quercus mongolica leaves. PLoS One 18 (8), e0289272. 10.1371/journal.pone.0289272 37611226 PMC10446833

[B79] Zeng X. Zou Y. Zheng J. Qiu S. Liu L. Wei C. (2023). Quorum sensing-mediated microbial interactions: mechanisms, applications, challenges and perspectives. Microbiol. Res. 273, 127414. 10.1016/j.micres.2023.127414 37236065

[B80] Zhang C. Gao Z. Shi W. Li L. Tian R. Huang J. (2020). Material conversion, microbial community composition and metabolic functional succession during green soybean hull composting. Bioresour. Technol. 316, 123823. 10.1016/j.biortech.2020.123823 32795866

[B81] Zhang Y. Gallant É. Park J. D. Seyedsayamdost M. R. (2022a). The small-molecule language of dynamic microbial interactions. Annu. Rev. Microbiol. 76, 641–660. 10.1146/annurev-micro-042722-091052 35679616 PMC10171915

[B82] Zhang Z. Mei X. He Z. Xie X. Yang Y. Mei C. (2022b). Nicotine metabolism pathway in bacteria: mechanism, modification, and application. Appl. Microbiol. Biotechnol. 106 (3), 889–904. 10.1007/s00253-022-11763-y 35072735

[B83] Zhang Q. Yang S. Yang Z. Zheng T. Li P. Zhou Q. (2023a). Effects of a novel microbial fermentation medium produced by Tremella aurantialba SCT-F3 on cigar filler leaf. Front. Microbiol. 14, 1267916. 10.3389/fmicb.2023.1267916 37808308 PMC10556473

[B84] Zhang Q. Zheng T. Yang Z. Yang S. Cai W. Li P. (2023b). Analysis of the structure and metabolic function of microbial community in cigar tobacco leaves in agricultural processing stage. Front. Microbiol. 14, 1230547. 10.3389/fmicb.2023.1230547 37637128 PMC10448963

[B85] Zhang M. Guo D. Wang H. Wu G. Shi Y. Zhou J. (2024a). Analyzing microbial community and volatile compound profiles in the fermentation of cigar tobacco leaves. Appl. Microbiol. Biotechnol. 108 (1), 243. 10.1007/s00253-024-13043-3 38421433 PMC10904427

[B86] Zhang Q. Huang Y. An H. Yang S. Lei J. Wang Y. (2024b). The impact of gradient variable temperature fermentation on the quality of cigar tobacco leaves. Front. Microbiol. 15, 1433656. 10.3389/fmicb.2024.1433656 39735193 PMC11672604

[B87] Zhang T. Liao Z. Bi J. Li Z. Liu Y. Liu Y. (2025a). Multi-omics analysis reveals microbial community succession and aroma enhancement mechanisms during spontaneous oak-barrel fermentation of chardonnay wine. Food Res. Int. 221 (Pt 1), 117249. 10.1016/j.foodres.2025.117249 41606853

[B88] Zhang W. Deng Q. Zhu B. Xiao D. Chen Q. Pan H. (2025b). Improving the quality of low-grade tobacco by enzymatic treatment and Co-fermentation with yeast and lactic acid bacteria. Appl. Biochem. Biotechnol. 197 (1), 613–630. 10.1007/s12010-024-05007-0 39207681

[B89] Zhang Y. Tian L. Wu D. Kong L. Zhang S. Gao Q. (2026). The role of bacterial-fungal interactions in cigar tobacco fermentation: insights from community dynamics and physicochemical correlations. BMC Microbiol. 10, 242. 10.1186/s12866-025-04567-3 41519720 PMC12990565

[B90] Zhao L. Zhu C. Gao Y. Wang C. Li X. Shu M. (2012). Nicotine degradation enhancement by Pseudomonas stutzeri ZCJ during aging process of tobacco leaves. World J. Microbiol. Biotechnol. 28 (5), 2077–2086. 10.1007/s11274-012-1010-9 22806029

[B91] Zhao S. Li Y. Liu F. Song Z. Yang W. Lei Y. (2024). Dynamic changes in fungal communities and functions in different air-curing stages of cigar tobacco leaves. Front. Microbiol. 15, 1361649. 10.3389/fmicb.2024.1361649 38567079 PMC10985334

[B92] Zhao L. Qian L. Guo L. Lin J. Song W. Yu X. (2025). Enhancing cellulose and hemicellulose degradation in wheat straw composting by inoculation with glycomyces: key factors and microbial community dynamics. Environ. Technol. 46 (16), 3036–3046. 10.1080/09593330.2025.2451782 39833992

[B93] Zheng T. Zhang Q. Li P. Wu X. Liu Y. Yang Z. (2022a). Analysis of microbial community, volatile flavor compounds, and flavor of cigar tobacco leaves from different regions. Front. Microbiol. 13, 907270. 10.3389/fmicb.2022.907270 35756070 PMC9231593

[B94] Zheng T. Zhang Q. Wu Q. Li D. Wu X. Li P. (2022b). Effects of inoculation with acinetobacter on fermentation of cigar tobacco leaves. Front. Microbiol. 13, 911791. 10.3389/fmicb.2022.911791 35783443 PMC9248808

[B95] Zhong W. Zhu C. Shu M. Sun K. Zhao L. Wang C. (2010). Degradation of nicotine in tobacco waste extract by newly isolated Pseudomonas sp. ZUTSKD. Bioresour. Technol. 101 (18), 6935–6941. 10.1016/j.biortech.2010.03.142 20434329

[B96] Zhong Y. Sorensen P. O. Zhu G. Jia X. Liu J. Shangguan Z. (2022). Differential microbial assembly processes and co-occurrence networks in the soil-root continuum along an environmental gradient. Imeta 1 (2), e18. 10.1002/imt2.18 38868564 PMC10989781

[B97] Zhou J. Liu J. Wang D. Ruan Y. Gong S. Gou J. (2024). Fungal communities are more sensitive to mildew than bacterial communities during tobacco storage processes. Appl. Microbiol. Biotechnol. 108 (1), 88. 10.1007/s00253-023-12882-w 38194134 PMC13496886

